# Phenformin-Induced Apoptosis: A Potential Mechanism for Cervical Cancer Cell Inhibition

**DOI:** 10.3390/ijms27114941

**Published:** 2026-05-29

**Authors:** Gehad M. Subaiea, Yernar Amangelsin, Kamila Sagatbekova, Ahmed A. Katamesh, Sameer A. Alkubati, Ahmed A. Alobaida, Hanan Abdelmawgoud Atia, Nasrin E. Khalifa, Thamir M. Alshammari, Reezal Ishak, Mohamad Aljofan

**Affiliations:** 1 Department of Pharmacology and Toxicology, College of Pharmacy, University of Ha’il, Hail 55473, Saudi Arabia; g.subaiea@uoh.edu.sa (G.M.S.); h.soliman@uoh.edu.sa (H.A.A.); 2 Medical and Diagnostic Research Center, University of Ha’il, Hail 55473, Saudi Arabia; a.katamsh@uoh.edu.sa (A.A.K.); s.alkubati@uoh.edu.sa (S.A.A.); a.alobaida@uoh.edu.sa (A.A.A.); n.aldirdiri@uoh.edu.sa (N.E.K.); 3 Department of Biomedical Sciences, School of Medicine, Nazarbayaev University, Astana 010000, Kazakhstan; yernar.amangelsin@alumni.nu.edu.kz (Y.A.); kamila.sagatbekova@nu.edu.kz (K.S.); 4 Department of Pharmaceutics, College of Pharmacy, University of Ha’il, Hail 55473, Saudi Arabia; 5 Department of Medical Surgical Nursing, College of Nursing, University of Ha’il, Hail 55473, Saudi Arabia; 6 Department of Pharmacy Practice, College of Pharmacy, Jazan University, Jazan 45142, Saudi Arabia; talshammari@jazanu.edu.sa; 7 Institute of Medical Science Technology (UniKL MESTECH), Universiti Kuala Lumpur, A1-1, Jalan TKS 1, Taman Kajang Sentral, Kajang 43000, Selangor, Malaysia; reezal@unikl.edu.my; 8 National Laboratory Astana, Astana 010000, Kazakhstan

**Keywords:** apoptosis, cervical cancer, drug repurposing, phenformin

## Abstract

Phenformin, a representative of the biguanides class was previously used for treatment of type 2 diabetes and discontinued due to a risk of causing lactic acidosis, has shown promising anticancer activity in numerous studies. Since many types of cancer arise and proliferate due to dysregulation in apoptotic or autophagic pathways, this study aimed to assess the underlying anticancer effects of phenformin in terms of these two processes. We initially set out to examine the antiproliferative effects of phenformin on multiple cancer cell lines including breast, pancreatic, cervical cancers, hepatocellular carcinoma and malignant melanoma. Subsequently, the expression of apoptosis and autophagy related proteins was measured in the cervical cancer cell lines found to be the most susceptible to antiproliferative effects of phenformin. Additionally, the ability of phenformin to potentiate the antitumor effect of resveratrol and vistusertib was assessed. Phenformin increased the expression of pro-apoptotic factor, Bax, and lowered the level of anti-apoptotic protein, Bcl-2. Hence, it was proposed that phenformin promotes antiproliferative activity by inducing apoptosis. Our findings demonstrate that phenformin decreases the proliferation of various cancer cell lines in a dose-dependent manner and may have an ability to increase the autophagic flux in cervical cancer cells. Our findings demonstrate that phenformin decreases the proliferation of various cancer cell lines in a dose-dependent manner potentially by inducing apoptosis.

## 1. Introduction

Apoptosis is an important mechanism of removal of impaired or non-essential cells and is essential for maintaining cellular homeostasis [1,2]. Proper signaling during apoptosis is crucial to ensure the delicate balance between the survival and death of cells [3]. An example of this is the imbalance of pro- and anti-apoptotic proteins within the Bcl-2 family [4], which can lead to apoptosis dysregulation and promote the survival of cancer cells [5]. It has been noted that the primary cause of this may be the elevated expression of anti-apoptotic proteins, the reduced expression of pro-apoptotic proteins, or a synergistic interaction between the two [6]. Additionally, apoptosis may be activated via death receptor-dependent pathways [7].

The potential use of biguanides as anticancer agents has gained significant attention in recent years. Metformin has been proposed to lower both the risk and incidence of cancer among diabetic patients [8,9]. Phenformin is more potent than metformin in terms of anticancer activity due to a presence of a hydrophobic benzene ring in its structure, which may facilitate cellular uptake [10]. According to Vancura et al. [11], metformin may mediate its antitumor activity by affecting the mitochondrial electron transport chain (ETC), AMP-activated kinase (AMPK), and mammalian target of rapamycin (mTOR), which play critical roles in cellular metabolism. Due to structural similarities, metformin and phenformin may act through similar mechanisms. Based on previous studies, several potential mechanisms for the activity of phenformin in inhibiting cancer cell growth have been proposed.

**Figure 1:**
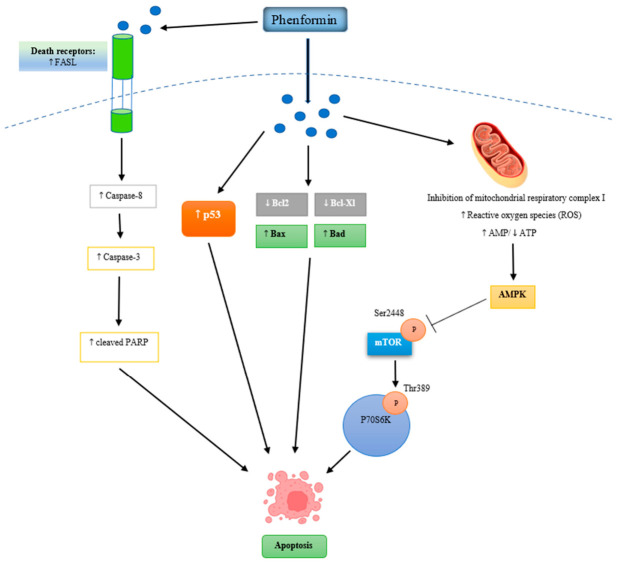
The anticancer mechanism of phenformin. Figure was made by authors in Biorender based on previous studies [13,17,18,19,20].

Previous studies have proposed multiple mechanisms by which phenformin may inhibit cancer cell growth including apoptosis [12] and autophagy [13], suppression of loss of contact inhibition [14], inhibition of mitochondrial complex I [15], inhibition of epithelial–mesenchymal transition [15] and G1 cell cycle arrest [16]. Figure 1 illustrates the proposed mechanisms by which phenformin induces apoptosis in cancer cells through multiple converging signaling pathways. Phenformin triggers intrinsic mitochondrial stress by inhibiting mitochondrial respiratory complex I, leading to increased reactive oxygen species (ROS) production and a reduction in cellular ATP levels [17]. These metabolic changes activate AMPK [13], which in turn suppresses mTOR signaling, resulting in downstream inhibition of the p70S6K pathway and promotion of apoptotic processes [18]. In parallel, phenformin modulates key apoptotic regulators by increasing p53 activity and shifting the balance of Bcl-2 family proteins toward a pro-apoptotic state, characterized by decreased Bcl-2 and Bcl-XL expression and increased Bax and Bad expression. Additionally, phenformin enhances extrinsic apoptosis signaling through upregulation of death receptor pathways (FASL), leading to activation of caspase-8, which subsequently activates caspase-3 and promotes PARP cleavage [19].

Together, these intrinsic and extrinsic pathways converge to drive programmed cell death (apoptosis), highlighting the multifaceted anticancer effects of phenformin.

Phenformin showed antiproliferative effects on several cancer cell lines, including breast [19], ovarian [17], cholangiocarcinoma [20], head and neck squamous cell carcinoma [18], and kidney [21] cancer cell lines. Since phenformin was withdrawn from the market due to concerns over lactic acidosis in diabetes patients, studies of its mechanism of action in cancer cells are rare, despite higher potency compared to metformin.

We hypothesize that apoptosis could be a primary mechanism underlying phenformin’s anticancer activity. Thus, our objective was to evaluate the potential anti-tumorigenic effects of phenformin in different cancer cell lines and evaluate the effect of phenformin on apoptosis-associated protein expression on the most susceptible cell line by performing Western blot quantification of apoptosis related proteins.

## 2. Results

### 2.1. Phenformin Treatment Results in a Dose-Dependent Decrease in Proliferation of Multiple Types of Cancer Cells

**Figure 2:**
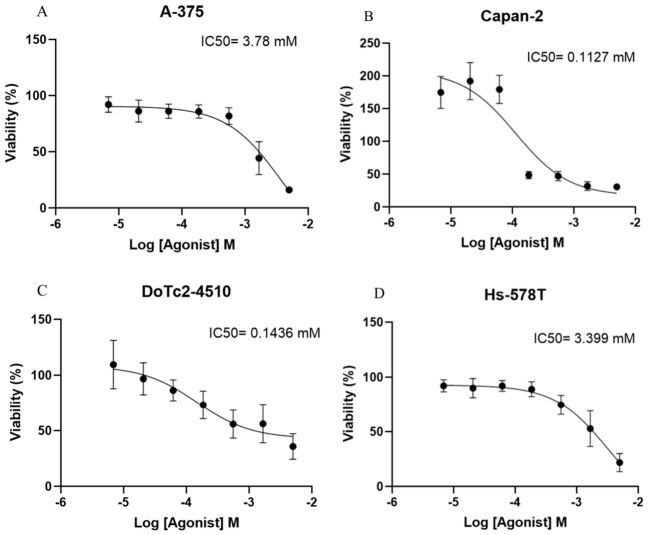

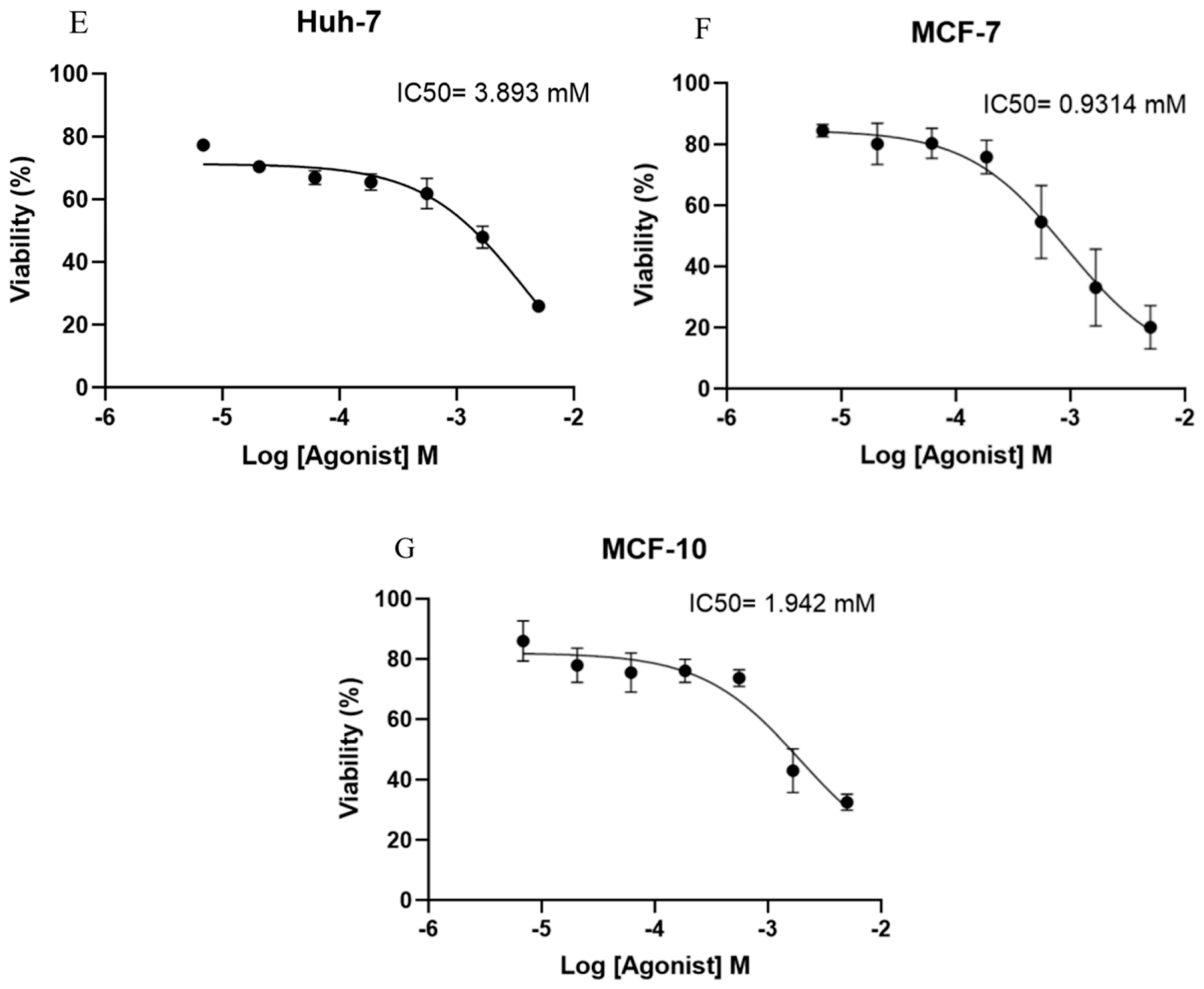
Antiproliferative activity of phenformin on various cell lines. (**A**–**G**) Antiproliferative activity of phenformin on A-375, Capan-2, DoTc2-4510, Hs-578T, Huh-7, MCF-7 and MCF-10 cell lines. The findings are presented as the mean ± SE of triplicate samples from three different trials.

The selected cell lines: DoTc2-4510, Hs-578T, MCF-7, A-375, Huh-7, Capan-2, and the non-tumorigenic MCF-10 were exposed to a range of phenformin concentrations (5 mM to 0.007 mM) for 48 h. A marked reduction in cellular proliferation in all cell lines was observed at concentrations of 5 and 1.67 mM (Figure 2A–G). In contrast, lower concentrations (0.55, 0.18, 0.062, 0.021, and 0.007 mM) did not significantly affect cell viability in A-375, Hs-578T, Huh-7, and MCF-10 cells. The IC_50_ values for these cell lines were calculated as 3.78, 3.39, 3.89, and 1.94 mM, respectively. It should be noted that reductions in MTT signal may reflect mitochondrial metabolic suppression rather than reduced cell number or increased cell death. Based on these findings, DoTc2-4510 was identified as the most sensitive cell line according to MTT-derived IC_50_ values and was selected for subsequent mechanistic studies.

### 2.2. Upregulation of Pro-Apoptotic and Downregulation of Anti-Apoptotic Proteins upon Phenformin Treatment

**Figure 3:**
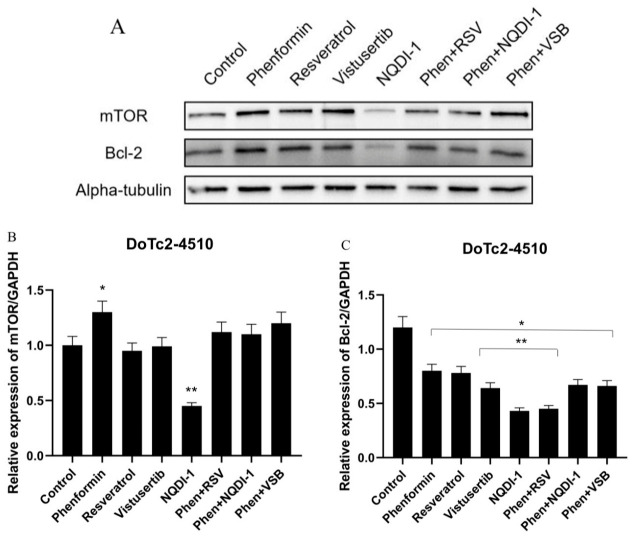

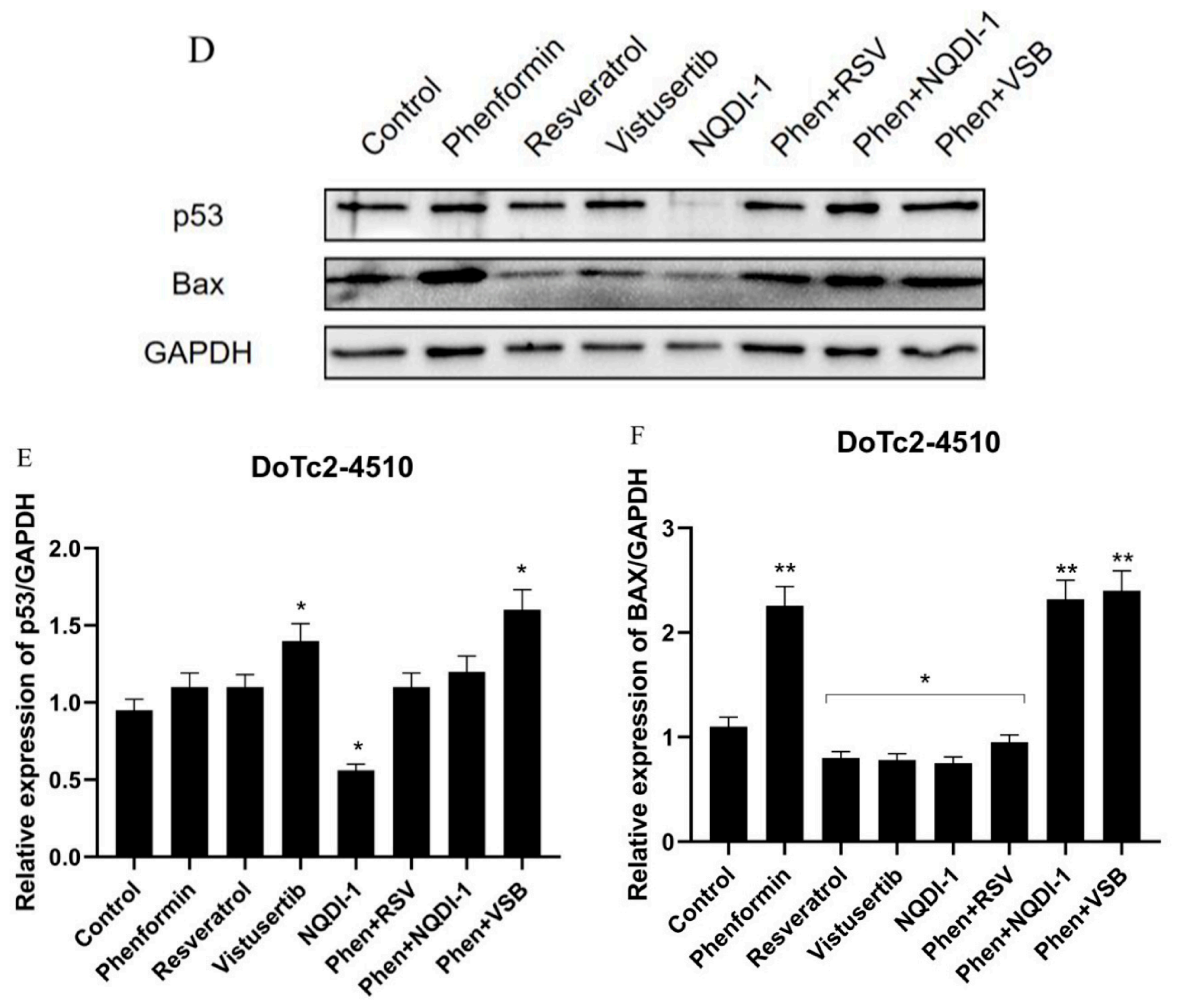
The effect of phenformin on the expression of apoptosis-related proteins. (**A**–**C**) Western blot analysis of mTOR and Bcl-2. (**D**–**F**) Western blot analysis of p53 and BAX. The Western blot analyses pointed out that the expression of anti-apoptotic Bcl-2 was decreased, whereas the expression of pro-apoptotic BAX was increased in phenformin only treated group (**C**,**F**). Data are presented as mean ± SD. * *p* < 0.05, ** *p* < 0.01 versus control.

The western blot analysis was performed to determine the effect of drug treatments on proteins that play a crucial role in cell survival and apoptosis, such as mTOR, p53, Bax, and Bcl-2 (Figure 3A–F). Phenformin significantly increased the level of mTOR expression compared to control, whereas (Figure 3A). There was no significant change in mTOR level between resveratrol and phenformin plus resveratrol treatment. The protein level was lower when treated with NQDI-1 alone. However, when NQDI-1 was combined with phenformin the level of mTOR was increased by roughly 2 fold. These results suggest that phenformin increases the expression of total mTOR protein level. All studies drugs and their combinations significantly decreased the level of anti-apoptotic Bcl-2 protein (Figure 3C). Treatment with phemformin did not result in a statistically significant decrease p-53 expression (Figure 3E). Phenformin increased the expression of pro-apoptotic BAX protein more than 2-fold both as a signle treatment and in combinaiton with NQDI-1 and vistusertib (Figure 3F).

## 3. Discussion

There was a dose-dependent reduction in the proliferation of Capan-2 pancreatic cancer cells. Interestingly, at a lower concentration of 0.062 mM, phenformin treatment appeared to increase the proliferation of these cells. These findings suggest that low concentrations of phenformin may paradoxically stimulate Capan-2 pancreatic cancer cell growth. We hypothesize that at sub-cytotoxic doses, phenformin may enhance nutrient uptake, such as glucose, thereby transiently promoting cell proliferation. Nevertheless, further studies are required to confirm and elucidate the underlying mechanisms of this phenomenon.

In cervical (DoTc2-4510) and breast (MCF-7) cancer cells, the dose-dependent reduction in cellular proliferation was more pronounced than in the other studied cell lines. The decrease in MCF-7 cell viability under phenformin treatment has also been reported previously [16]. The IC_50_ values of phenformin for DoTc2-4510 and MCF-7 cell lines were 0.1436 mM and 0.9314 mM, respectively, indicating greater potency against the cervical cancer cell line. These results suggest that DoTc2-4510 cells are particularly sensitive to phenformin. To the best of our knowledge, this is the first report of the antiproliferative effect of phenformin on the DoTc2-4510 cell line. The IC50 values obtained for phenformin were in the millimolar range, which may raise concerns regarding pharmacological feasibility and clinical translation. While phenformin exhibited significant biological effects in vitro, the concentrations required to achieve these effects may exceed physiologically tolerable levels in clinical settings. Further studies involving optimized delivery strategies, combination approaches, or structural modifications may be necessary to improve therapeutic applicability.

We investigated the effect of phenformin on apoptosis induction in combination with drugs, such as resveratrol, vistusertib, and NQDI-1. Resveratrol is a herbal substance with antioxidative, anti-inflammatory, and anticancer properties [22]. Some plants, including peanuts, soy, and grapes, can be a reference product for resveratrol extraction [22]. According to Ashrafizadeh [23], the antiproliferative activity of resveratrol is based on the activation of cellular apoptosis. Resveratrol induces apoptosis of cancer cells by activating pro-apoptotic proteins and p53, as well as inhibiting anti-apoptotic proteins and PI3K/Akt/mTOR signaling pathway [23]. Moreover, resveratrol can activate AMPK by producing ROS, thereby reducing phosphorylation of mTOR [24]. Vistusertib is a substance that inactivates both mTORC1 and mTORC2 protein complexes [25]. It promotes cancer cell death through inhibition of the PI3K/mTOR signaling pathway. ASK1, apoptosis signal-regulating kinase, is a protein kinase that plays a key role in cell differentiation and apoptosis induction [26]. NQDI-1 is a pharmacological inhibitor of ASK1 [27]. Hao et al. [28] have reported that the inhibition of ASK1 by NQDI-1 resulted in a significant reduction in the expression of ASK1 target proteins, including phosphorylated JNK and c-jun, p53, and caspase-3. Therefore, NQDI-1 can inhibit cellular apoptosis by inactivating ASK1 protein kinase.

Previous studies indicate that phenformin activates AMPK and inhibits mTOR. However, in our experiments, total mTOR protein levels were increased following phenformin treatment. Similarly, mTOR levels were elevated in cells treated with resveratrol and vistusertib, both alone and in combination with phenformin. Previous studies on breast and ovarian cancer cells have reported that phenformin had no effect on the total expression of mTOR protein in breast cancer cells [17,18]. Fan et al. [29] investigated the effect of resveratrol to induce apoptosis of promyelocytic leukemia HL-60. The study finding also demonstrated that resveratrol activated AMPK and inhibited mTOR, thereby reducing the phosphorylation of p70S6K. Guichard et al. [30] investigated the antitumor effect of vistusertib on breast cancer cells. The results concluded that vistusertib inhibits both mTORC1 and mTORC2 proteins, thus reducing the phosphorylation of downstream target proteins, such as S6 and AKT. Notably, in these studies, total mTOR protein remained unchanged.

The aforementioned studies indicate that phenformin, resveratrol, and vistusertib possess the ability to inhibit the mTOR protein, while having no impact on the overall protein expression. However, our experiment produced different results, with the above-mentioned drugs upregulating mTOR expression. The discrepancy between our results and previous findings suggests that drug effects on mTOR expression may be cell line-dependent. To clarify the functional impact of phenformin on mTOR signaling, future studies should assess phosphorylated mTOR and downstream targets.

It is believed that the upregulation of p53 induces apoptosis. However, in our study, phenformin decreased the level of p53. The similar trend was observed in the study by Kuo et al., according to this study, the downregulation of p53 leads to the disruption of DEC1 (transcription factor), a downstream target gene of p53. The study findings suggest that phenformin induces apoptosis of cervical cancer cells by downregulating p53 and DEC, which was validated by increased cleaved PARP and caspase-3 levels [31].

Seo et al. [19] have investigated the effect of phenformin treatment on FaDu cells. According to their results, phenformin downregulated the levels of anti-apoptotic proteins, Bcl-2 and Bcl-Xl, and upregulated the level of pro-apoptotic proteins, Bad and Bax. Our findings align with this, as phenformin also decreased Bcl-2 and increased Bax in DoTc2-4510 cells, suggesting apoptosis induction via a mitochondria-dependent intrinsic pathway.

In a previous study on A498 kidney cancer cells by Zhu et al. [32], at different concentrations ranging from 7.5 to 30 μM galangin was not found to have any effect on overall mTOR expression, however the level of expression of phosphorylated mTOR (p-mTOR) was markedly decreased. Similarly, phenformin reduced the phosphorylation of mTOR at serine 2448 in a dose-dependent manner in prostate cancer cells [33]. These observations indicate that phenformin may regulate mTOR activity at the phosphorylation level rather than total protein abundance, which aligns with our findings showing unchanged total mTOR but potential functional inhibition.

## 4. Materials and Methods

### 4.1. Cell Culture

Human breast (MCF-7, Hs 578T), pancreatic (Capan-2), cervical (DoTc2-4510), hepatocellular carcinoma (HUH-7) and malignant melanoma (A-375) cell lines as well as a non-tumorigenic human mammary epithelial cell line (MCF-10) were maintained in Dulbecco’s modified Eagle’s medium (DMEM, Sigma, St. Louis, MO, USA) completed with 10% fetal bovine serum (FBS) and 1% penicillin–streptomycin. Cells were maintained under normal culture conditions in the incubator at 37 °C with 5% CO_2_ (Binder, Model CB-S 170, Tuttlingen, Germany).

### 4.2. Drug Preparation

Phenformin (Sigma, St. Louis, MO, USA) was dissolved in distilled water to make 100 mM stock solutions. Resveratrol (MedChemExpress, Monmouth Junction, NJ, USA), vistusertib (MedChemExpress, Monmouth Junction, NJ, USA), galangin (Sigma, St. Louis, MO, USA), and NQDI-1 (MedChemExpress, Monmouth Junction, NJ, USA) were dissolved in dimethyl sulfoxide (DMSO) to prepare 100 mM, 200 μM, 5 mM, and 5 mM stock solutions, respectively. These stock solutions were used in Western blot analysis.

### 4.3. MTT Assay

The MTT assay was used as a surrogate measure of cellular metabolic activity. All cells were grown in 96-well plates at a density of 3000 cells per well for 24 h. Afterwards, the cells were treated with various concentrations of phenformin ranging from 0.0068 mM to 5 mM for 48 h. Given that phenformin targets mitochondrial complex I, MTT readouts may be influenced by metabolic inhibition rather than direct effects on cell viability; therefore, results should be interpreted with caution. Each experiment was conducted in triplicate and repeated in three independent experiments (biological replicates) to ensure reproducibility.

### 4.4. Western Blotting

Cells were lysed in radio immunoprecipitation assay (RIPA) lysis buffer (50 mM Tris-HCl, pH 8.0, 150 mM NaCl, 1% Nonidet P-40, 0.5% sodium deoxycholate, 0.1% sodium dodecyl sulfate) with protease inhibitor cocktail (Roche, Basel, Switzerland). After sonication, samples were at 16,000× *g* for 20 min at a precooled centrifuge. Protein concertation was quantified using the Bicinchoninic Acid (BCA) assay kit (Thermo Fisher, Waltham, MA, USA) and analyzed by SDS-PAGE. The gel was then transferred to a polyvinylidene difluoride membrane (Bio-Rad, Hercules, CA, USA) using the wet transfer apparatus (Bio-Rad, Hercules, CA, USA). The membrane was blocked in 1% BSA in 1× PBS. Following the washing, membranes were incubated with primary antibodies Bax (1:500, Cusabio, Wuhan, China), Bcl-2 (1:500, Sigma, St. Louis, MO, USA), p53 (1:500, Sigma, St. Louis, MO, USA), mTOR (1:500, Sigma, St. Louis, MO, USA), α-tubulin (1:1000, Sigma, St. Louis, MO, USA), GAPDH (1:2000, Sigma, St. Louis, MO, USA) on a shaker for an hour at room temperature. Thereafter, the blots were visualized by ECL (Bio-Rad, Hercules, CA, USA).

## 5. Conclusions

In summary, our findings demonstrate that phenformin reduces MTT-based metabolic activity in DoTc2-4510 cervical cancer cells. Moreover, phenformin modulated the expression of apoptosis-related proteins, including downregulation of Bcl-2 and upregulation of Bax. However, due to the absence of functional validation assays, these findings should be interpreted as indicative of pathway modulation rather than definitive evidence of apoptosis induction. To date, this study represents the first report of phenformin activity in the DoTc2-4510 cervical cancer cell line. Further studies incorporating functional assays, multiple cell models, and clinically relevant dosing are required to validate and extend these findings.

## Data Availability

The original contributions presented in this study are included in the article. Further inquiries can be directed to the corresponding author.
